# Preface to ‘Green carbon for the chemical industry of the future’

**DOI:** 10.1098/rsta.2023.0274

**Published:** 2024-09-23

**Authors:** Graham J. Hutchings, C. Richard A. Catlow, Matthew Davidson, Matthew J. Rosseinsky, Charotte Williams

**Affiliations:** ^1^ Cardiff Catalysis Institute, School of Chemistry, Cardiff University, Cardiff CF24 4HQ, UK; ^2^ Centre for Sustainable and Circular Technologies, University of Bath, Claverton Down, Bath BA2 7AY, UK; ^3^ Department of Chemistry, University of Liverpool, Crown St, Liverpool L69 7ZD, UK; ^4^ Department of Chemistry, Inorganic Chemistry Laboratory, South Parks Road, Oxford OX1 3QR, UK

**Keywords:** green chemistry, green carbon, chemical industry

The chemical industry is an important industrial sector and is responsible for providing many of the materials that are essential for the well-being of society. Such materials include polymers, fertilizers, disinfectants and pharmaceuticals to name just a few important effect materials. It is estimated that over 100 000 chemicals are currently produced, each targeted to a specific application. At present, the greater majority of these chemicals are produced using fossil carbon, i.e. from natural gas, oil and coal. A large number of the chemicals are derived as by-products from oil refining, which is operated primarily to produce gasoline and diesel. Today, there is a growing awareness that anthropomorphic carbon dioxide emissions through the excessive use of fossil carbon resources are leading to global warming and therefore represent an existential threat to life on our planet. It was against this background that a Royal Society Discussion Meeting on the topic of *Green carbon for the chemical industry of the future* was held in London on 11–12 December 2023. The meeting was attended by over 150 delegates in a hybrid meeting with participants present online and in person. The topic for this discussion meeting was how can we replace fossil carbon with carbon derived from sustainable sources, which we denote as green carbon. The concept of green hydrogen is well recognized by society as hydrogen that is derived from water electrolysis using sustainable energy. It is the combination of green carbon with green hydrogen in new chemical processes that can provide the basis for the chemical industry of the future.

The papers collected in this special edition are based on some of the presentations at the discussion meeting. They represent a broad spectrum of topics that show how the new chemical industry can be transformed to offer a sustainable future, thereby ensuring that all the chemicals that make our everyday lives both viable and comfortable will still be available to us as we drive towards a net zero society.

The paper includes an article on ‘Green Carbon and the Chemical Industry of the Future’ by Roger Sheldon that sets out the scale of the challenge and the opportunities for the sector. When one evaluates the sustainability of carbon sources it is important to consider life cycle analysis and this key topic is addressed in a paper by Marcelle McManus on ‘Towards a unified carbon accounting landscape’. Many papers discuss the possible new pathways that will be needed to generate the chemical intermediates that will be required, as well as the possible new production routes for key chemicals such as methanol and ammonia. In addition, papers address the possibility of using photons or electrons to drive the new chemical processes rather than relying on the traditional methods using heat and pressure that are energy intensive.

On 8 May 2024, the Royal Society launched a Policy Briefing on this topic entitled ‘Catalysing change: defossilising the chemical industry’. In this report, three sources of sustainable green carbon were considered, namely waste polymers, biomass and carbon dioxide. The conclusion was that the chemical industry of the future would use a mix of all three, but without intervention, the transition to net zero for the chemical industry will take many decades. This is time we do not have and so it is important that we now actively pursue and address the challenges and exciting new opportunities presented by using sustainable green carbon in place of fossil carbon.

## Data Availability

This article has no additional data.

